# The Reliability of Classifications of Proximal Femoral Fractures with 3-Dimensional Computed Tomography: The New Concept of Comprehensive Classification

**DOI:** 10.1155/2014/359689

**Published:** 2014-12-25

**Authors:** Hiroaki Kijima, Shin Yamada, Natsuo Konishi, Hitoshi Kubota, Hiroshi Tazawa, Takayuki Tani, Norio Suzuki, Keiji Kamo, Yoshihiko Okudera, Ken Sasaki, Tetsuya Kawano, Yoichi Shimada

**Affiliations:** ^1^Department of Orthopedic Surgery, Akita University Graduate School of Medicine, 1-1-1 Hondo, Akita 010-8543, Japan; ^2^Akita Hip Research Group, Akita 010-8543, Japan

## Abstract

The reliability of proximal femoral fracture classifications using 3DCT was evaluated, and a comprehensive “area classification” was developed. Eleven orthopedists (5–26 years from graduation) classified 27 proximal femoral fractures at one hospital from June 2013 to July 2014 based on preoperative images. Various classifications were compared to “area classification.” In “area classification,” the proximal femur is divided into 4 areas with 3 boundary lines: Line-1 is the center of the neck, Line-2 is the border between the neck and the trochanteric zone, and Line-3 links the inferior borders of the greater and lesser trochanters. A fracture only in the first area was classified as a pure first area fracture; one in the first and second area was classified as a 1-2 type fracture. In the same way, fractures were classified as pure 2, 3-4, 1-2-3, and so on. “Area classification” reliability was highest when orthopedists with varying experience classified proximal femoral fractures using 3DCT. Other classifications cannot classify proximal femoral fractures if they exceed each classification's particular zones. However, fractures that exceed the target zones are “dangerous” fractures. “Area classification” can classify such fractures, and it is therefore useful for selecting osteosynthesis methods.

## 1. Introduction

Proximal femoral fractures are very common injuries [1–4]. However, there is no global consensus on the choice of treatment methods for proximal femoral fractures.

In most cases of proximal femoral fractures, good results are achieved by performing osteosynthesis [5, 6] or total hip replacement (femoral head replacement). However, in some of these types of fractures there is an increased risk of serious complications, such as pseudarthrosis or cutting out of the implant, unless the treatment methods are chosen very carefully [7–11].

Various classification methods (e.g., the AO classification [12]) have been used to identify such “dangerous” fractures and to choose appropriate therapeutic methods. In most cases, at first, femoral neck fractures or trochanteric fractures are distinguished. In the case of neck fractures, osteosynthesis or arthroplasty is chosen based on the degree of displacement or the stage in Garden's classification [13] after considering patient age or activities. When osteosynthesis is performed, the choice of the implant to use for osteosynthesis may be changed using Pauwels' classification [14], which evaluates instability from the angles of the main fracture lines.

In the case of trochanteric fractures, osteosynthesis is often chosen. However, many osteosynthesis methods have been reported, and because of this, a classification by the number of bone fragments on 3-dimensional computed tomography (3DCT) [15] came to be frequently used in choosing methods of osteosynthesis.

In addition, proximal femoral fractures broken near the border between the neck and the trochanteric part are said to be basicervical fractures (basal neck fractures) [12]. The classification of Pauwels [14] may be applied also for basicervical fractures, because some think that the angle of the main fracture line is important in such fractures. The AO classification [12], which is a systematic classification including neck fractures, basicervical fractures, and trochanteric fractures, is used worldwide.

However, the results vary greatly among examiners when fractures are classified using these classifications [16–20]. Previous studies have examined the reliability of only the AO classification and Garden's classification for neck fractures and of the Evans classification for trochanteric fractures. In these reports, the fractures were classified only with X-ray films. However, multidirectional reconstruction CT or 3DCT is now deployed at most hospitals due to recent progress in imaging technology. Therefore, treatment based on classification using CT will be necessary for proximal femoral fractures, but there have been no investigations of the reliability of classifications for proximal femoral fractures using 3DCT.

In addition, even if using an existing classification, proximal femoral fractures, which exceed each classification's particular zones, cannot be classified. However, such fractures that cross the specific zones are “dangerous” fractures that can easily lead to pseudarthrosis and cut-out of the implant.

Therefore, the reliability of some classifications of proximal femoral fractures when 3DCT was used was examined in detail. In addition, a new comprehensive classification for proximal femoral fractures (“area classification”), which can make up for the faults of the existing classifications and was useful for choosing treatment methods, was developed.

## 2. Materials and Methods

A total of 27 consecutive proximal femoral fracture cases seen at a single facility from June 2013 to July 2014 were investigated. Eleven orthopedic surgeons, who graduated from medical school 5–26 years earlier, evaluated the preoperative X-rays images, axial images of CT, and 3DCT images of these 27 cases. In this study, the AO classification [12], Garden's classification [13], 3DCT classification of trochanteric fractures [15], the classification of Pauwels [14], and the new “area classification” were used.

**Figure 1:**
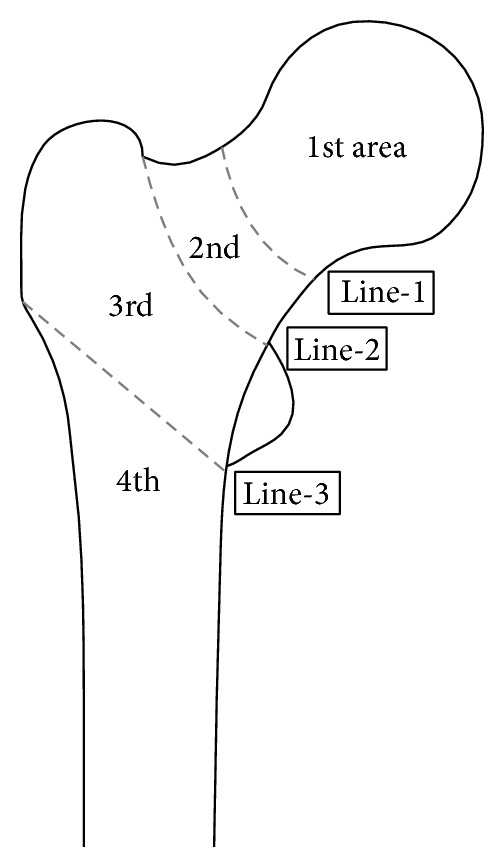
The boundary lines that separate areas in “area classification.” In “area classification,” the proximal femur is divided into 4 areas with 3 boundary lines; Line-1 is the center of the neck, Line-2 is the border between the neck and the trochanteric zone, and Line-3 is the line that links the inferior borders of the greater and lesser trochanters.

**Figure 2:**
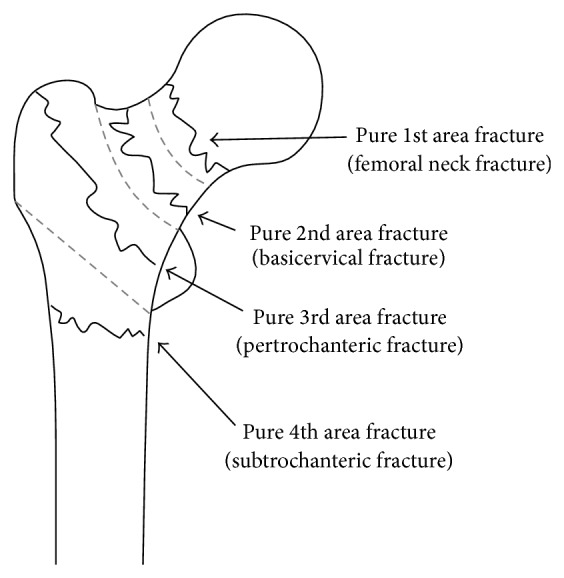
Pure type fractures in “area classification.” When there is a fracture in only the first area, the fracture is classified as a pure first area fracture, and so on. Thus, pure 1 type is the so-called neck fracture, pure 2 type is the so-called basicervical fracture, pure 3 type is the so-called trochanteric fracture, and pure 4 type is the so-called subtrochanteric fracture.

**Figure 3:**
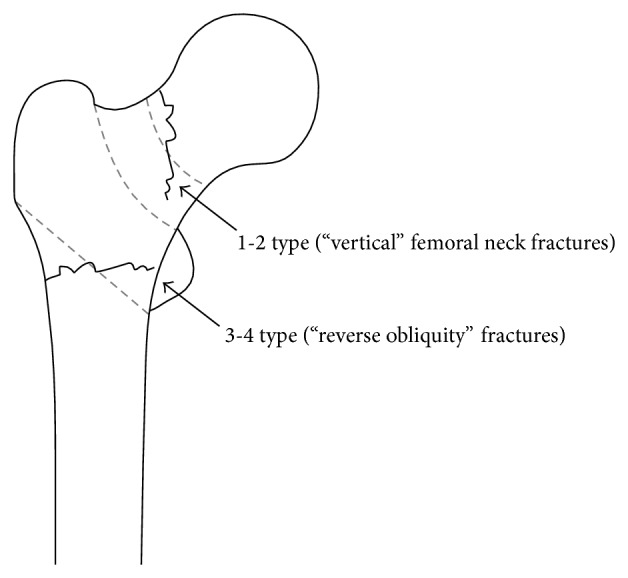
The “dangerous” fractures that exceed each classification's particular zones. For example, 1-2 type fractures are vertical fractures that extend to the basal neck from the neck. 3-4 type fractures are equivalent to type 2 of the Evans classification; their instability is greater than general trochanteric fractures.

**Figure 4:**
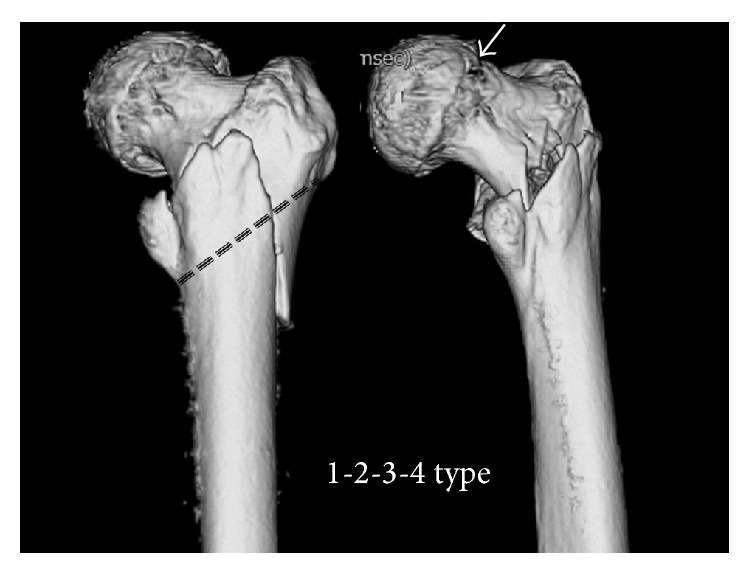
A fracture that penetrates all areas. A fracture is detected (white arrow).

In “area classification,” the proximal femur was divided into 4 areas with 3 boundary lines; Line-1 is the center of the neck, Line-2 is the border between the neck and the trochanteric zone, and Line-3 is the line that links the inferior borders of the greater and lesser trochanters (Figure 1). If a fracture was only in the first area, the fracture was classified as a pure first area fracture, and if a fracture was present in the first and second areas, the fracture was classified as a 1-2 type fracture. In the same way, fractures were classified as pure 2, pure 3, 2-3, 3-4, 1-2-3, and so on. In this classification, the so-called neck fractures, basicervical fractures, trochanteric fractures, and subtrochanteric fractures were defined by the boundary lines (Figure 2). In addition, “area classification” can classify the fractures that crossed the zones (Figures 3 and 4).

In other words, pure 1 type is the so-called neck fracture, pure 2 type is the so-called basicervical fracture, pure 3 type is the so-called trochanteric fracture, and pure 4 type is the so-called subtrochanteric fracture (Figure 2). The 1-2 type is the fracture whose fracture line ranges from the neck to the basal neck, the 2-3 type is the fracture whose fracture line ranges from the basal neck to the trochanteric zone, and the 1-2-3 type is the fracture whose fracture line ranges from the neck to the trochanteric zone (Figure 3).

In addition, a fracture such as a type 2 of the Evans classification [21] is classified as a 3-4 type fracture because the fracture line ranges from the third area to the fourth area (Figure 3). However, even if a fracture line of a fragment of the lesser trochanter alone was in the second area or the fourth area, it was classified as a pure 3 type, because the fracture line of a lesser trochanteric fragment is not the main fracture line. However, when the fracture line of the big fragment including the lesser trochanter entered into the fourth area outside the center of Line-3, this was classified as involving the fourth area.

First, 11 orthopedic surgeons classified all 27 cases using the AO classification [12] and the “area classification.” If the fracture was classified as a neck fracture, it was also classified using Garden's classification [13]. If the fracture was classified as a trochanteric fracture, the fracture was also classified using the 3DCT classification [15]. The classification of Pauwels is usually used only for a neck fracture, but, in this study, it was applied to all 27 cases, classified only based on the angle of the main fracture line.

The reliability of each classification was evaluated using Fleiss' Kappa coefficient [22].

## 3. Results

The patients' average age was 76 years (55–95 years); there were 6 male and 21 female cases. The reliabilities (Fleiss' Kappa coefficients) of the AO classification (A1–C3), the AO classification in detail, Garden's classification, the 3DCT classification of trochanteric fractures, the Pauwels classification, and “area classification” were 0.4751, 0.1964, 0.3682, 0.4404, 0.1796, and 0.5154, respectively. The AO classification and 3DCT classification had to be applied with the classification list. However, the classification list was not necessary for “area classification” to be used.

## 4. Discussion

This study is the first to investigate reliability when proximal femoral fractures were classified using 3DCT. Furthermore, in this study, the reliability of “area classification,” which we devised, was the highest, and orthopedic surgeons with greatly varied years of experience classified proximal femoral fractures using various classifications. Withother classifications, the fractures cannot be classified if the fractures exceed each classification's particular zones. However, the fractures that exceed the target zones are “dangerous” fractures that increase the risk of serious complications such as pseudarthrosis or cutting out of the implant. “Area classification” can classify such fractures. Therefore, “area classification” is useful when choosing the method of osteosynthesis.

In recent years, due to the severe osteoporosis of elderly persons or due to high energy injuries such as traffic accidents, proximal femoral fractures are aggravated. Therefore, complex fractures, which step over the zone targeted for various classifications that have been used previously, have increased. Such cases cannot be evaluated using conventional classifications; thus, a combination of classifications is used. However, proximal femoral fractures are not treated as several multiple fractures, because the solidity of the whole proximal femur must be considered. Therefore, comprehensive classification related to treatment methods or clinical results was necessary. The “area classification” that we developed is one attempt to address this issue.

Furthermore, most conventional classifications are used with X-ray films. However, with recent progression of imaging technology, multidimensional CT or 3DCT is deployed at most hospitals and is useful for diagnosis or choice of treatment method for proximal femoral fractures as well. However, there have been no reports about the reliability of the conventional classification when many orthopedic surgeons used such CT images. As a result of having tried it in this study for the first time, the reliabilities of conventional classifications were not high, and it became clear that the reliability of “area classification” was the highest, because this classification is very comprehensive and very simple.

Furthermore, the advantage of “area classification” is that it can be used with familiar conventional classifications. In “area classification,” pure 1 type is a neck fracture, pure 2 type is a basicervical fracture, pure 3 type is a trochanteric fracture, and pure 4 type is a subtrochanteric fracture. Therefore, conventional classifications can be used if the fractures are classified by “area classification” as a pure type. If the fractures are classified by “area classification” as extending to multiple areas, such fractures need special treatment. For instance, 1-2 type fractures are vertical fractures that extend to the basal neck from the neck; thus, rotation instability with shear stress must be considered (Figure 3). For example, 3-4 type fractures are equivalent to type 2 of the Evans classification [21]; thus, they have greater instability than general trochanteric fractures (Figure 3). In this way, instability can be evaluated using “area classification,” and the treatment methods are chosen very carefully.

However, even “area classification” did not have a sufficiently high Fleiss' Kappa coefficient. There are several reasons for this. First, there were few cases. Second, when there are multiple fractures in this area, the observer may not recognize other fractures if he pays attention to only one fracture line. Third, because the pelvis is also in the 3DCT image, it may be hard to see the femur. Fourth, the definition of Line-1 is somewhat vague. Fifth, it was necessary to turn the 3DCT images or to evaluate thin slices of the axial CT images because Line-2 is covered by the greater trochanter on the posterior side. However, the 3DCT images that the observers evaluated could not be turned in all directions. In addition, the number of slices of axial CT images was insufficient. Sixth, with respect to Line-3, it was slightly more complicated to handle when a fragment of the lesser trochanter alone occurred. Therefore, it is thought that higher reliability is provided if the observers use 3DCT images that can be turned in every direction after having removed the pelvis. In addition, the definitions of the linesmay be improved when “area classification” is used by many orthopedists. However, even if the reliability was not very high, it is one of the greatest advantages that the use of “area classification” is connected to the evaluation of instability and careful choice of treatment method.

The limitation of this study is that clinical results were not examined. The “area classification” that we devised has higher reliability than other classifications, and, theoretically, no cases are unclassifiable. Thus, in the future, we can investigate the relationship between the classification results when “area classification” is used and the choice of treatment methods or the clinical results of proximal femoral fractures. In other words, the results of this investigation, which involved many facilities that agreed to use this classification, could result in better treatment for proximal femoral fractures. The current study is thus the first step leading to further investigation.

## 5. Conclusions

The reliability of “area classification” was the highest when 11 orthopedists classified proximal femoral fractures using 3DCT. Withother classifications, proximal femoral fractures cannot be classified when the fractures exceed each classification's particular zones. However, the fractures that exceed the target zones are “dangerous” fractures. “Area classification” can classify such fractures. Therefore, “area classification” is useful in choosing the methods of treatment.

## References

[1] Karagas M. R., Lu-Yao G. L., Barrett J. A., Beach M. L., Baron J. A. (1996). Heterogeneity of hip fracture: age, race, sex, and geographic patterns of femoral neck and trochanteric fractures among the US elderly. *American Journal of Epidemiology*.

[2] Bjorgul K., Reikeras O. (2007). Incidence of hip fracture in south eastern Norway: a study of 1730 cervical and trochanteric fractures. *International Orthopaedics*.

[3] Johansen A., Wakeman R., Boulton C., Plant F., Roberts J., Williams A. (2013). *National Hip Fracture Database: National Report 2013*.

[4] Queally J. M., Harris E., Handoll H. H., Parker M. J. (2014). Intramedullary nails for extracapsular hip fractures in adults. *Cochrane Database of Systematic Reviews*.

[5] Kraus M., Krischak G., Wiedmann K., Riepl C., Gebhard F., Jöckel J. A., Scola A. (2011). Clinical evaluation of PFNA and relationship between the tip-apex distance and mechanical failure. *Unfallchirurg*.

[6] Mereddy P., Kamath S., Ramakrishnan M., Malik H., Donnachie N. (2009). The AO/ASIF proximal femoral nail antirotation (PFNA): a new design for the treatment of unstable proximal femoral fractures. *Injury*.

[7] Muhm M., Hillenbrand H., Danko T., Weiss C., Ruffing T., Winkler H. (2013). Frühkomplikationsrate bei hüftgelenknahen Frakturen. *Der Unfallchirurg*.

[8] Bonnaire F., Weber A., Bösl O., Eckhardt C., Schwieger K., Linke B. (2007). “Cutting out” in pertrochanteric fractures—problem of osteoporosis?. *Unfallchirurg*.

[9] Bojan A. J., Beimel C., Taglang G., Collin D., Ekholm C., Jönsson A. (2013). Critical factors in cut-out complication after gamma nail treatment of proximal femoral fractures. *BMC Musculoskeletal Disorders*.

[10] Hsueh K.-K., Fang C.-K., Chen C.-M., Su Y.-P., Wu H.-F., Chiu F.-Y. (2010). Risk factors in cutout of sliding hip screw in intertrochanteric fractures: an evaluation of 937 patients. *International Orthopaedics*.

[11] Al-Munajjed A. A., Hammer J., Mayr E., Nerlich M., Lenich A. (2008). Biomechanical characterisation of osteosyntheses for proximal femur fractures: helical blade versus screw. *Studies in Health Technology and Informatics*.

[12] Muller M. E., Allgower M. (1991). Appendix A. The comprehensive classification of fractures of long bones. *Manual of Internal Fixation: Techniques Recommended by the AO-ASIF Group*.

[13] Garden R. S. (1961). Low-angle fixation in fractures of the femoral neck. *The Journal of Bone & Joint Surgery. British Volume*.

[14] Pauwels F. (1935). *Der schenkelhalsbruch: Ein mechanisches Problem*.

[15] Nakano T. (2006). Understanding of femoral trochanteric fracture in aged and the suggestion of classification with 3DCT. *Monthly Book Orthopaedics*.

[16] Blundell C. M., Parker M. J., Pryor G. A., Hopkinson-Woolley J., Bhonsle S. S. (1998). Assessment of the AO classification of intracapsular fractures of the proximal femur. *Journal of Bone and Joint Surgery*.

[17] Bjørgul K., Reikerås O. (2002). Low interobserver reliability of radiographic signs predicting healing disturbance in displaced intracapsular fracture of the femoral neck. *Acta Orthopaedica Scandinavica*.

[18] Thomsen N. O. B., Jensen C. M., Skovgaard N., Pedersen M. S., Pallesen P., Soe-Nielsen N. H., Rosenklint A. (1996). Observer variation in the radiographic classification of fractures of the neck of the femur using Garden's system. *International Orthopaedics*.

[19] Andersen E., Jorgensen L. G., Hededam L. T. (1990). Evans' classification of trochanteric fractures: an assessment of the interobserver and intraobserver reliability. *Injury*.

[20] Gehrchen P. M., Nielsen J. O., Olesen B. (1993). Poor reproducibility of Evans' classification of the trochanteric fracture: assessment of 4 observers in 52 cases. *Acta Orthopaedica Scandinavica*.

[21] Evans E. M. (1949). The treatment of trochanteric fractures of the femur. *The Journal of Bone & Joint Surgery. British Volume*.

[22] Siegel S., Castellan N. J. (1988). *Nonparametric Statistics for the Behavioral Sciences*.

